# Full-length cDNAs from chicken bursal lymphocytes to facilitate gene function analysis

**DOI:** 10.1186/gb-2004-6-1-r6

**Published:** 2004-12-23

**Authors:** Randolph B Caldwell, Andrzej M Kierzek, Hiroshi Arakawa, Yuri Bezzubov, Jolanta Zaim, Petra Fiedler, Stefan Kutter, Artem Blagodatski, Diyana Kostovska, Marek Koter, Jiri Plachy, Piero Carninci, Yoshihide Hayashizaki, Jean-Marie Buerstedde

**Affiliations:** 1 Institute of Molecular Radiobiology, GSF, Ingolstädter Landstrasse 1, D-85764 Neuherberg, Germany; 2 Laboratory of Systems Biology Institute of Biochemistry and Biophysics, Polish Academy of Sciences, Pawinskiego 5a, 02-106 Warszawa, Poland; 3 School of Biomedical and Molecular Sciences, University of Surrey, Guildford GU2 7XH, UK; 4 Academy of Sciences of the Czech Republic, Institute of Molecular Genetics, 16637 Praha, Czech Republic; 5 Laboratory for Genome Exploration Research Project, Genomic Sciences Center and Genome Science Laboratory, RIKEN Tsukuba Institute, 3-1-1 Koyadai, Tsukuba, Ibaraki 305-0074, Japan; 6 Genome Exploration Research Group, RIKEN Genomic Sciences Center, RIKEN Yokohama Institute, 1-7-22 Suehiro-cho, Tsurumi-ku, Yokohama, Kanagawa, 230-0045, Japan

## Abstract

A large number of cDNA inserts were sequenced from a high-quality library of chicken bursal lymphocyte cDNAs. Comparisons to public gene databases indicate that the cDNA collection represents more than 2,000 new, full-length transcripts. This resource defines the structure and the coding potential of a large fraction of B-cell specific and housekeeping genes whose function can be analyzed by disruption in the chicken DT40 B-cell line.

## Abstract 2

This article reports a cDNA collection representing more than 2000 new, full-length transcripts from a high-quality cDNA library.

## Background

Large-scale genomic and cDNA sequencing projects have revealed thousands of new genes whose open reading frames (ORFs) are highly conserved during vertebrate evolution, but whose precise cellular functions remain unclear. Although functional analysis by gene disruption is possible after transfection of murine embryonic stem cells and the breeding of knockout mice [1], these whole-animal studies are laborious and expensive. If the mutant phenotype can be distinguished in cell culture, the chicken B-cell line DT40 is a valid alternative to murine knockouts because of its high ratio of targeted gene integration [2-4]. Additional advantages of DT40 are tightly regulated conditional gene-expression systems for the analysis of essential genes [5-7] and the ability to study genetic interactions by the stepwise modification of multiple loci [8] and marker recycling [7].

The recent release of the chicken genome sequence [9] greatly benefits the DT40 research community. For the first time, the entire genome can be searched for sequences that are conserved during vertebrate evolution and whose function might be clarified after genetic modification in DT40. However, *in silico *gene structure prediction methods have a high error rate and often do not correctly annotate the intron-exon structure of genes. Only full-length cDNAs unambiguously define the boundaries of the transcription units within whole-genome assemblies and cloned full-length cDNAs are also of immense practical value to complement mutant phenotypes and artificially express the encoded protein [10]. For these reasons, many genome sequencing projects in higher eukaryotes have been complemented by large-scale efforts to obtain a maximum number of full-length cDNAs [11,12]. Although relatively large expressed sequence tag (EST) databases from bursal lymphocytes [13] and other tissues have been described [14], relatively few chicken cDNA sequences had been deposited in the public databases.

Here we describe a project to sequence and characterize a large number of full-length cDNAs from bursal lymphocytes. The corresponding genes are likely to be expressed in DT40 and this should facilitate their analysis by targeted gene modifications. In combination with the recently released cDNAs from other tissues [15], the bursal cDNAs will be a valuable resource for many laboratories working with the chicken as a model organism.

## Results and discussion

### Generation of bursal cDNA sequences

**Figure 1:**
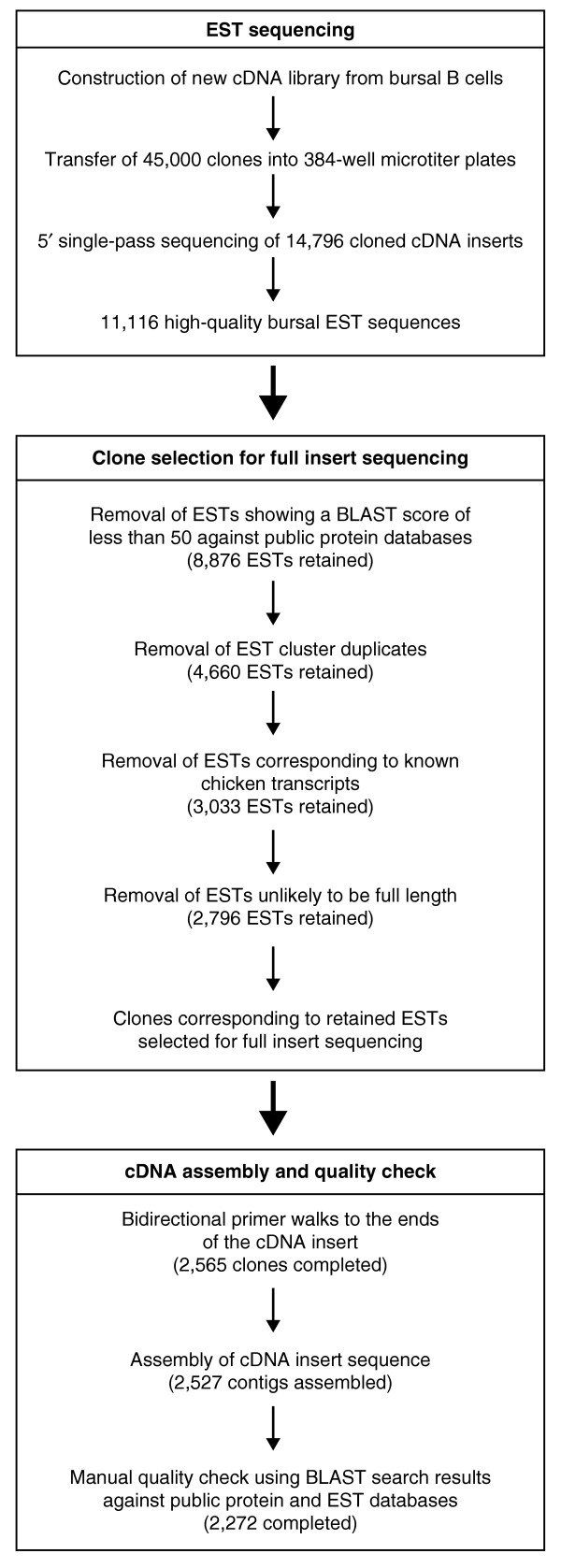
Outline of full-length bursal cDNA production.

The overall strategy for producing the greatest possible number of new full-length cDNAs expressed in bursal lymphocytes is outlined in Figure 1. We previously described a cDNA library of bursal lymphocytes, but it contained only a low number of full-length cDNA clones [13]. It was therefore decided to synthesize a new cDNA library, called 'riken1', using the biotinylated cap trapper method which is optimized to generate a large percentage of full-length cDNA inserts [16]. To assess the quality of the library and guide the selection of clones for full insert sequencing, the 5' ends of over 14,000 clone inserts were sequenced. BLAST [17] searches against the public protein databases indicated that about 80% of the 11,116 high-quality ESTs obtained showed significant homology to existing entries and more than 80% of these extended further upstream than the methionine start codon of their homologs in the databases. This indicated that the riken1 library indeed contains an extraordinary high percentage of full-length cDNA inserts. Only clones whose ESTs showed significant BLAST matches against the public protein databases and covered the methionine start codon of their homolog were considered for full-length sequencing, as evolutionarily conserved genes are of highest interest for the DT40 research community. The remaining ESTs were clustered to remove duplicates corresponding to the same gene. In addition, ESTs corresponding to already known chicken genes in the public databases were removed.

The plasmids corresponding to the remaining 2,796 ESTs were chosen for full insert sequencing by bidirectional primer walks. Once the end of the walks had been reached, the sequences of the full-length cDNA inserts were assembled. From the BLAST search results the most likely methionine start codon was assigned to each sequence. About 15% of the cDNA sequences showed evidence for premature frameshifts in the form of short ORFs and stretches of conserved sequence in a different frame further 3'. If overlapping ESTs were found in the public databases, the cDNA sequences were edited to correct the likely reverse transcription error, otherwise these sequences were discarded.

### Length distribution and GC content

**Figure 2:**
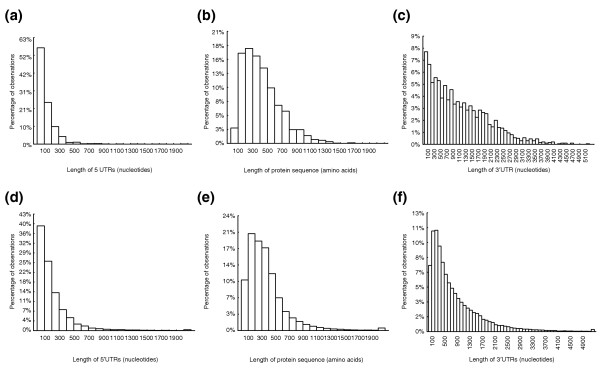
Comparison of the length distributions of bursal cDNAs and public database sequences. The length distributions of 5' UTRs from **(a) **bursal cDNAs and **(d) **5' UTR sequences present in UtrDB. The length distribution of annotated ORFs from **(b) **bursal cDNAs and **(e) **UniProt database sequences. The length distributions of 3' UTRs from **(c) **bursal cDNAs and **(f) **3' UTR sequences present in UtrDB. Lengths of UTR sequences are given in nucleotides; lengths of ORFs in amino acids.

A total of 2,272 high-quality chicken full-length cDNA clones were sequenced and assembled, manually annotated with respect to their likely translation start codon and deposited both at The Bursal Transcript Database website [18] and in the public databases. The lengths of the proteins encoded by the annotated ORFs were compared with the lengths of UniProt [19] database entries and the lengths of the untranslated region (UTR) sequences were compared with the lengths of known vertebrate UTRs available from the UtrDB collection [20] (Figure 2). The distributions obtained for the bursal cDNAs closely resemble those calculated for known sequences. Most of the 5' UTRs have lengths in the range of 100 base-pairs (bp) [21], a value conserved in diverse taxonomic classes. The length distribution of 3' UTRs is much broader, with a significant number of long sequences exceeding 1 kilobase (kb). The similarity between the length distributions observed for the collection presented here and those sequences stored in public databases suggests that most of our sequences are full-length cDNAs with correctly annotated start codon positions.

The most remarkable feature noted in the analysis of 5' UTRs of the bursal cDNAs is a very high GC content (67%). This supports the observation that the GC content of 5' UTRs is particularly high in warm-blooded species [22]. On the other hand, the percentage of GC base-pairs in 3' UTRs of the bursal cDNAs (41%) is close to the value observed for database sequences (42%). The ORFs of the bursal full-length cDNAs contain 49% GC base-pairs.

### Analysis of start codon context

The accurate prediction of the translation start codon remains difficult and in some cases our annotations remain tentative. Sequences surrounding the translation start codons are not random and in mammals match the consensus GCCRCCaugG (where aug is the start codon and R is either A or G) [23]. The most conserved nucleotides in the consensus are a purine, usually A, at position-3 and G at position 4. It has also been observed that a large fraction of 5' UTRs contain AUG codons upstream of the translation start site, but these codons are unlikely to be flanked by the consensus sequence [21].

A detailed analysis shows that the riken1 collection of cDNA sequences contains 4,406 AUG codons upstream of the annotated translation start codons in 2,218 of the bursal cDNAs. Nine hundred one of these alternative start codons were in the same reading frame as the annotated ORF. An in-frame stop codon within the 5' UTR region was present downstream of 501 of these 901 alternative start codons. The total number of ORFs present in 5' UTR regions of riken1 cDNAs was 1,289.

**Figure 3:**
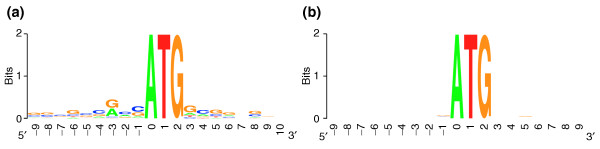
ATG codon context in the riken1 cDNA sequences. **(a) **Sequence logo of annotated start codon context. **(b) **Sequence logo of the context of ATGs found upstream of the annotated start codons. Figure created using WebLogo [35].

We have checked whether the context of the annotated AUG start codons differs from the context of the alternative upstream AUG sequences of the bursal cDNAs. We therefore extracted 10-bp long sequences surrounding the annotated start codons and the alternative upstream AUGs and visualized sequence variability using the sequence logo software [24] (Figure 3). The annotated start codons closely match the consensus, but the alternative upstream AUG codons do not exhibit flanking nucleotide preferences. This provides further evidence that the ORFs in our collection are correctly annotated.

### Similarity to predicted Ensembl transcripts and UniProt protein sequences

**Figure 4:**
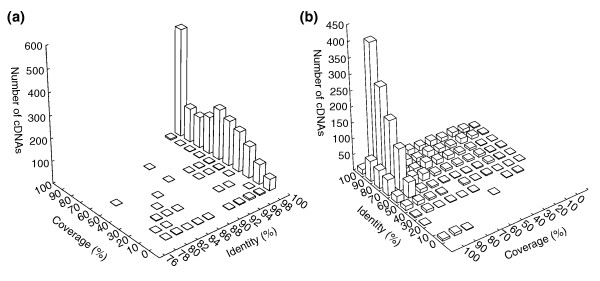
Statistics of BLAST searches. **(a) **BLASTN search of full-length cDNAs against chicken transcripts predicted by Ensembl. Note that only 494 cDNAs are identical or nearly identical with predicted transcripts. Therefore, the remaining 1,777 experimentally determined full-length cDNAs may be used to improve the gene annotation of chicken genomic DNA. **(b) **BLASTP searches of annotated ORFs against the UniProt database of protein sequences.

All full-length cDNAs were compared to the collection of transcripts predicted from the chicken genome sequence by the Ensembl system [25]. The transcripts were downloaded before the Ensembl team used our collection of full-length bursal cDNA sequences to improve transcript predictions. Distribution of the percent identity and coverage of the best BLASTN alignments are shown in Figure 4a. Only 494 of the chicken full-length transcripts matched predicted mRNAs with a length coverage greater than 90%. This is not surprising taking into account that computational prediction of untranslated regions, based on the genome sequence alone, is very difficult, if not impossible. However, there were also significant differences between sequenced and predicted cDNAs within ORF regions. There are 1,463 sequences in which either the 5' or the 3' end of the ORF was not covered by predicted transcripts. In most cases (1,106), the discrepancy concerned the 5' end. The statistics presented above and summarized in Table 1 indicate that our collection of full-length cDNA sequences may be used to significantly improve the annotations of more than 1,400 chicken genes. This analysis is further supported by the mapping of bursal serial analysis of gene expression (SAGE) tags to Ensembl transcripts and the genome sequence [26].

Figure 4b shows the distribution of the percent identity and coverage statistics of the BLASTP comparison of the proteins encoded by the bursal cDNAs to the UniProt collection of protein sequences. In most cases (1,524), the proteins encoded by riken1 cDNAs were almost fully covered in the alignments (more than 90% coverage) and showed a high percentage identity (greater than 70%) to known protein sequences.

When compared to available chicken ESTs or cDNAs in the public databases, some of the bursal cDNAs showed significant structural differences most likely due to differential transcript processing. In addition, the bursal cDNA collection has been used to define a large number of intragenic single-nucleotide polymorphisms (SNPs) [27].

### Functional domain assignment

All full-length cDNAs were compared to the Pfam database [28], which stores sequence profiles representing functional domains and the 10 most frequently occurring domains are shown in Table 2. Subsequently, we have used the Gene Ontology (GO) [29] annotation of Pfam domains provided by the InterPro [30] database to assign functional descriptors to the domains detected in our sequences. It is important to note that the assignment of a GO term to a given cDNA sequence indicates only the presence of a functional domain rather than an orthologous relationship to other genes annotated by the term. Determination of orthologous relationships is best done at the level of whole-genome comparisons and is therefore beyond the scope of this study. This classification will be valuable for the selection of candidate genes for further analysis in DT40, but it is unlikely to be representative for the whole chicken genome because only a selected subset of cDNAs expressed in bursal cells were chosen for sequencing.

Tables 3 and 4 list the assignments of GO molecular function and biological process descriptors to functional domains detected in riken1 full-length cDNAs. The most frequent molecular function associated with a domain is 'ATP binding' (166 cDNAs) assigned to the protein kinase domain and other domains such as the AAA ATPase family, ABC transporters and others. The 64 DNA-binding proteins represented in our collection include RNA polymerase II, C5-cytosine-specific DNA methylase and 19 proteins exhibiting transcription factor activity according to biological process annotation. GTP-binding proteins are involved in translation initiation (eIF-2-gamma), cell-cycle regulation (Septin 5) and regulation of transport from the endoplasmatic reticulum to the Golgi apparatus.

There are 22 full-length cDNAs in our collection containing Pfam domains annotated by the GO term 'molecular function unknown'. Experimental information concerning the molecular mechanisms of action is very sparse or nonexistent for proteins sharing these evolutionarily conserved domains. Highly similar human proteins exist for the chicken proteins, an example being the human protein BM02. Taking into account the ease of targeted genome modification and availability of numerous functional assays, the DT40 cell line is an attractive model system to provide first insights into the functions of the evolutionarily conserved domains described above.

### Bursal Transcript database

All the full-length cDNA sequences are stored within the Bursal Transcript database [18]. This database links the previously published EST data with the new cDNAs and can be searched by keyword or by using BLAST. Browsing of functional categories is also available as dynamically generated web pages link the bursal cDNAs to Ensembl, UniProt, Pfam and to GO data. To highlight gene expression differences between DT40 and bursal cells, the bursal cDNAs are also linked to SAGE data from both of these types of cells [26].

## Conclusions

The cDNAs from bursal lymphocytes represent one of the largest full-length cDNA collections in the chicken, comprising about one third of all currently available, experimentally verified transcripts and will be of general interest to researchers using the chicken as an experimental model as well as to the poultry industry. The resource has already been integrated with the chicken genome sequences to build a unigene catalog [9], to define the nature and frequency of intragenic chicken strain polymorphisms [27] and to develop a chicken gene microarray for gene-expression profiling (B. Wong, T. Makeev and C. Davies, unpublished data). However, the main beneficiary of the full-length cDNAs is the DT40 research community. Although the release of the genome sequence has greatly simplified the identification of candidate genes for disruption and the design of the knockout constructs, it is still not a trivial task to predict the ORFs as well as 3' and 5' UTRs without cDNA sequences. Other uses are the expression of the cDNAs *in vitro *or for complementation of mutant DT40 phenotypes with the added convenience that the cDNA sequences are not only known, but also available as cloned pieces of fully sequenced DNA.

## Materials and methods

### Construction of the riken1 cDNA library and 5' EST sequencing

The riken1 library was synthesized from mRNA of 2-week-old CB strain bursal lymphocytes using the biotinylated cap trapper method [16,31]. The resulting phage library was converted into pKS-derived plasmids and individual clones were then selected on ampicillin-containing agarose plates. About 45,000 colonies were picked and transferred into 384-well microtiter plates to prepare a permanent clone stock. Plasmids from 14,976 of the arrayed clones were sequenced on an Applied Biosystems automated sequencer using a primer that anneals to the plasmid backbone upstream of the 5' end of the cDNA inserts (see [18] for details of the cloning vector sequence). The ABI sequencing files were processed as described previously [13]. About 5% of the riken1 clones contained an insert sequence which was 100% identical to the GenBank entry AJ277662, annotated as a human genomic fragment including the *LMO1 *locus. This sequence was present as a stuffer of the lambda vector used for the library construction and the clones containing it were removed from further analysis. In total, the 5' single-pass sequencing of 14,976 clones yielded 11,116 high-quality ESTs of the riken1 library.

### Selection of clones for full-length insert sequencing

BLAST searches against the 'All non-redundant GenBank CDS' database showed that approximately 80% of the 5' EST sequences matched GenBank entries with a score of at least 50. This score threshold was chosen because it allowed us in most cases to align the putative start codon of the query sequence to the EST. These sequences were chosen and clustered [32] to remove duplicates. In addition, all sequences matching chicken entries in the public databases with a score of over 300 were not considered further. The BLAST results of all remaining sequences were manually inspected and only those sequences which covered the methionine start codon of their closest match in the public databases were retained. In the end, the cDNA inserts corresponding to 2,796 ESTs were chosen for full insert sequencing.

### Full-length insert sequencing

Sufficient plasmid template for numerous sequencing reactions was prepared from the clones corresponding to the selected ESTs. All plasmids were then sequenced with a primer complementary to a plasmid sequence 3' of the cDNA insertion site. Subsequently, custom-made 20-mer primers based on available sequences were used for sequencing until the 3' and 5' ends of the cDNA inserts were reached. All sequences were processed as described previously [13], except that a routine of manual proofreading and editing of the chromatograms within the Staden pregap program was implemented to increase the quality of the base calling and to decrease the failure rate of the next primer walk. The FOUNTAIN software [32] was extended to automatically design primers in 96-well format suitable for these walks. The primers were positioned to give an average of a 70-bp overlap between sequences. Once both ends of the insert were reached by the primer walks, the Staden gap4 program [33] was used to produce a double-stranded consensus of the cDNA insert. A total of 2,565 high-quality cDNA contigs were assembled for further analysis.

### Quality check and correction of frameshifts in the cDNA sequences

The integrity of the conserved ORF within each assembled cDNA sequence was manually examined by inspecting BLAST search results against the public protein and EST databases. To facilitate this task, a new EstSet module was added to FOUNTAIN [32]. The user interface displays the sequence of the cDNA insert together with its three possible translations and its BLAST search results against the public protein and EST databases. On the basis of this information a likely methionine start codon can be assigned to the cDNA. Around 15% of the cDNA sequences showed evidence of an artificial frameshift in the form of suspicious BLAST matches in two or more ORFs, presumably due to errors in the reverse transcription process. These sequences were compared to other *Gallus gallus *ESTs from the public databases. If the short ORF could be corrected by adopting the sequence of an overlapping EST, the cDNA sequence was edited. The type of editing was recorded and the corresponding riken1 clone was annotated as likely to be defective. In total, 293 cDNAs were removed because either a likely artificial frameshift could not be corrected by using sequences of overlapping ESTs or they contained multiple stop codons in all three reading frames or they showed evidence for unspliced introns. All cDNA clones are freely available upon request to the corresponding author and their sequences have been submitted to the EMBL public database (accession numbers AJ719267-AJ721138 and AJ851370-AJ851825).

### Analysis of the start codon context

The sequences surrounding the annotated start codons (10 bp upstream and downstream) were exported and submitted for information content visualization by the WebLogo software [24]. Subsequently, we have exported the ± 10 bp context of every ATG codon located upstream of the annotated start of the coding sequence. These sequences were also submitted to analysis by WebLogo software.

### Sequence-similarity searches and functional class annotation

The riken1 cDNAs were compared with the collection of predicted chicken transcripts downloaded from the Ensembl ftp site [34] using the BLASTN program. BLASTP software was used to compare translated ORFs with the protein sequences stored in the UniProt database. Functional domains were assigned by comparing riken1 cDNAs with sequence profiles representing Pfam domains. This comparison was performed with RPSBLAST software (e-value cut-off of 10^-6^) run on the binary database files downloaded from the National Center for Biotechnology Information (NCBI). Functional classes were assigned according to Pfam to GO mapping provided by the InterPro database. The XML information exchange standard was used to interface the BLAST program outputs with the FOUNTAIN package.

## Figures and Tables

**Table 1:** Comparison of riken1 cDNAs and cDNAs predicted by Ensembl

	Number of riken1 cDNAs	Percentage of the total number of riken1 cDNAs
More than 90% identity and more than 90% coverage	494	22
More than 90% identity and more than 70% coverage	808	36
More than 90% identity and more than 50% coverage	1258	55
Less than 90% identity	19	0.8
3' or 5' UTR of riken1 cDNA uncovered	1,463	64
5' UTR of riken1 cDNA uncovered	1,106	49

**Table 2:** The 10 most frequently occurring Pfam domains in chicken full-length cDNAs

Pfam ID	Description	Number of occurrences
PF00069	Protein kinase domain	66
PF00076	RRM_1: RNA recognition motif	46
PF00071	Ras family	32
PF00025	Arf: ADP-ribosylation factor family	23
PF00169	PH: pleckstrin homology domain.	22
PF00271	Helicase_C, Helicase conserved C-terminal domain	20
PF00018	SH3 (Src homology 3) domain	18
PF00651	BTB or POZ domain present in some of zinc finger proteins	18
PF00270	DEAD, DEAD/DEAH box helicase	18
PF00004	AAA, ATPase family associated with various cellular activities	14

**Table 3:** The 10 molecular function GO terms most frequently assigned to chicken cDNAs

GO ID	Description	Number of occurrences*
GO:0005524	ATP binding	166
GO:0004672	Protein kinase activity	66
GO:0003677	DNA binding	64
GO:0005525	GTP binding	57
GO:0005515	Protein binding	37
GO:0016491	Oxidoreductase activity	27
GO:0005554	Molecular function unknown	22
GO:0003700	Transcription factor activity	19
GO:0008565	Protein transporter activity	18
GO:0008270	Zinc ion binding	17

*Number of cDNAs containing domain annotated by the GO term.

**Table 4:** The 10 biological process GO terms most frequently assigned to chicken cDNAs

GO ID	Description	Number of occurrences*
GO:0006468	Protein amino acid phosphorylation	66
GO:0006355	Regulation of transcription, DNA-dependent	39
GO:0006508	Proteolysis and peptidolysis	38
GO:0007264	Small GTPase mediated signal transduction	33
GO:0006412	Protein biosynthesis	29
GO:0006118	Electron transport	28
GO:0006886	Intracellular protein transport	26
GO:0007165	Signal transduction	18
GO:0005975	Carbohydrate metabolism	17
GO:0006457	Protein folding	11

*Number of cDNAs containing domain annotated by the GO term.
